# Bayesian Models for N-of-1 Trials

**DOI:** 10.1162/99608f92.3f1772ce

**Published:** 2022-09-08

**Authors:** Christopher Schmid, Jiabei Yang

**Affiliations:** 1 Department of Biostatistics, School of Public Health, Brown University, Providence, Rhode Island, United States of America

**Keywords:** meta-analysis, multilevel model, inflammatory bowel disease, personalized medicine, Markov chain Monte Carlo, posterior inference

## Abstract

We describe Bayesian models for data from N-of-1 trials, reviewing both the basics of Bayesian inference and applications to data from single trials and collections of trials sharing the same research questions and data structures. Bayesian inference is natural for drawing inferences from N-of-1 trials because it can incorporate external and subjective information to supplement trial data as well as give straightforward interpretations of posterior probabilities as an individual’s state of knowledge about their own condition after their trial. Bayesian models are also easily augmented to incorporate specific characteristics of N-of-1 data such as trend, carryover, and autocorrelation and offer flexibility of implementation. Combining data from multiple N-of-1 trials using Bayesian multilevel models leads naturally to inferences about population and subgroup parameters such as average treatment effects and treatment effect heterogeneity and to improved inferences about individual parameters. Data from a trial comparing different diets for treating children with inflammatory bowel disease are used to illustrate the models and inferences that may be drawn. The analysis shows that certain diets were better on average at reducing pain, but that benefits were restricted to a subset of patients and that withdrawal from the study was a good marker for lack of benefit.

## Media Summary

N-of-1 trials (or personalized trials) are randomized trials for comparing the effects of two or more interventions on an outcome on a single individual. Bayesian methods are natural for statistical analysis of data from N-of-1 trials because they give straightforward interpretations of the probability of treatment outcomes that supplement the information coming from the trial with information from external sources and the individual’s own subjective beliefs. These posterior probabilities update the individual’s state of knowledge about their own condition after their trial. Bayesian models can also easily incorporate specific characteristics of N-of-1 data such as trend, carryover, and autocorrelation and offer flexibility of implementation. By combining the data from a collection of N-of-1 trials, Bayesian multilevel models also lead naturally to inferences about average treatment effects and heterogeneity of effects across subgroups in the population and to improved inferences about effects in individuals.

This article reviews the basics of Bayesian inference and models for both single N-of-1 trials and collections of N-of-1 trials sharing the same research questions and data structures. Data from a trial comparing different diets for treating children with inflammatory bowel disease are used to illustrate the models and inferences that may be drawn. The analysis shows that certain diets were better on average at reducing pain, but that benefits were restricted to a subset of patients and that withdrawal from the study was a good marker for lack of benefit.

## 1. Introduction

Randomized controlled trials (RCTs) are generally considered the gold standard tool for assessing the average efficacy of health care interventions in specified patient populations. Clinicians, however, are focused on treating individual patients in particular settings that may differ from the context in which the RCTs have been conducted. Because trials focus on average efficacy, results may not apply to an individual patient who requires treatment (Duan et al., 2013).

N-of-1 trials are a type of single-case experimental design in which two or more interventions are applied multiple times in a randomized order to an individual participant. Interventions are typically crossed over multiple times and multiple measurements may be made during each intervention period. The resulting set of measurements provides an estimate of treatment efficacy for the individual, generating truly personalized evidence (Schork, 2015).

N-of-1 trial designs may also be personalized so that individuals design their own trials evaluating interventions and outcomes of interest to them in a manner of their choosing. For example, the PREEMPT trial compared the use of N-of-1 trials to usual care for patients with chronic musculoskeletal pain (Kravitz et al., 2018). Participants randomized to the N-of-1 arm set up their own trials comparing two treatments of their choice and using between two and four intervention periods of one or two weeks on each treatment. They then scored themselves daily on five patient-reported outcomes. Such trials could usefully be termed ‘personalized trials.’

When participants use similar trial designs, though, it may be possible to combine them analytically using a multilevel model in a meta-analysis to provide evidence of efficacy for groups of individuals and even improve estimates for the individuals themselves (Gelman & Hill, 2006)(Zucker et al., 1997). For example, (Kaplan et al., 2022) compared two special diets, each assigned for two eight-week periods to 54 children with inflammatory bowel disease (IBD). (Marcus et al., 2022) assessed the impact of triggers for atrial fibrillation by having individuals test triggers over a 6-week trial. Each trigger was tested for three randomly chosen weeks and the number of atrial fibrillation episodes was compared to the number in the other three weeks in which no trigger was tested.

The purpose of this article is to describe ways to analyze both individual and combined N-of-1 trial data using Bayesian inference. Bayesian models provide a flexible framework for constructing and computing complex models that incorporate information both from the data at hand and from relevant external evidence, thus facilitating principled and transparent inference that aids decision-making.

The Bayesian framework provides several advantages for analyzing N-of-1 data. First, it allows participants, both patients and clinicians, to use a prior distribution to combine their own subjective assessment of treatment efficacy with the experimental data to come up with a posterior assessment needed to make a decision. Second, knowing the entire joint posterior distribution of all model parameters enables making direct statements about the probability of a variety of scientific hypotheses such as that one treatment is better than another by a certain amount and about composite statements of interest such as the chance that one treatment is better on each of two outcomes. It also eliminates the need to rely on asymptotic normality for drawing inferences as with maximum likelihood estimation. When trials are aggregated, inferences can be easily drawn about each individual borrowing strength using information from other individuals. Finally, missing data may be treated as random quantities so that imputation follows directly from their posterior distribution.

For the remainder of the article, we shall assume that the conditions under which N-of-1 trials are appropriate hold: substantial therapeutic uncertainty about treatment, heterogeneous treatment effects, a stable chronic condition, short-acting treatments with rapid onset, no carryover of treatment effect, and measurable outcomes that are easy to collect and whose levels return to baseline after each treatment period (n.d.-a).

The article is organized as follows. Section 2 introduces models for a continuous outcome in a single trial that incorporate treatment effects, trend, autocorrelation, and carryover. Section 3 discusses Bayesian models and various considerations that go into their computation, interpretation, and evaluation with application to the N-of-1 model. In Section 4, we extend the modeling to a collection of individuals, discussing how a multilevel framework permits estimating common parameters as well as improving estimation of individual parameters. Section 5 applies the model to data from a recent series of N-of-1 trials evaluating the effects of two different diets on a pain outcome for children with inflammatory bowel disease. In Section 6, we discuss extensions of models to other types of data such as discrete outcomes and networks of trials. Section 7 covers some issues relevant to practical use of N-of-1 trials, such as presenting results to participants and implementation before a short summary in Section 8.

Before moving on, we point out some useful general references for readers desiring more detail on Bayesian inference (Gelman et al., 2013), meta-analysis (Schmid, Stijnen, et al., 2020b), and N-of-1 trials (n.d.-b).

## 2. Model for a Single Trial

### 2.1. Treatment Effects

Let us begin by specifying a simple design for a single N-of-1 trial comparing K treatments. Often K=2. Let $Y_{j}$ denote the $j^{th}$ measurement of a continuous outcome with corresponding treatment $A_{j}$ taking on values k=1,…,K. Each treatment is given for a certain period of time and the treatment periods are randomized. For example, if daily treatments are randomized in weekly blocks so that all treatments in a given week are the same, $A_{1},\ldots ,A_{7}$ would have the same value as would $A_{8},\ldots ,A_{14}$. In the simplest model, we ignore any effects of time and write

(2.1) $$Y_{j}=m+\sum_{k\neq 1}\delta_{k}I\left(A_{j}=k\right)+\epsilon_{j}$$

(2.2) $$\epsilon_{j}∼N\left(0,\sigma^{2}\right)$$

where m is the mean outcome for the reference treatment $A_{j}=1$ and $\delta_{k}$ is the difference between the mean outcomes on treatment $A_{j}=k$ and the reference treatment for k=2,…,K. This is called a contrast-based model because it is formulated in terms of the contrasts between each treatment and the reference treatment. Equivalently, we could write an arm-based model in terms of the K treatment arms and their means $m_{k}$ as

(2.3) $$Y_{j}=\sum_{k}m_{k}I\left(A_{j}=k\right)+\epsilon_{j}.$$

The treatment effect comparing treatment k to k' is estimated by $m_{k}-m_{k'}$ for k,k'∈{1,…,K,k≠k'}. The data include J measurements $y=\{y_{1},\ldots ,y_{J}\}$; the parameters are $\Theta =\{m,\sigma^{2},\delta_{2},\ldots ,\delta_{K}\}$ for the contrast-based model and $\Theta =\{\sigma^{2},m_{1},\ldots ,m_{K}\}$ for the arm-based model.

### 2.2. Trend

As an N-of-1 trial evolves over time, underlying forces may lead to changes in the outcome independent of treatment effects. Such changes can often be captured by smooth trends, the simplest of which is linear trend in which the outcome changes as a linear function of time. We can incorporate trend by adding a linear term to Equation 2.1 as

(2.4) $$Y_{j}=m+\sum_{k\neq 1}\delta_{k}I\left(A_{j}=k\right)+\beta t_{j}+\epsilon_{j}$$

where $t_{j}$ is the time at which measurement j is taken and β is the change in the outcome per unit time. We can also add this linear trend to Equation 2.3.

Trend can also be nonlinear. Introducing effects for each treatment period creates a step function. This might be reasonable if one believes that the individual learns over time and starts from a new baseline in each period. Cyclical trend is also realistic for conditions that may wax and wane and may be modeled with sinusoidal or spline functions.

### 2.3. Autocorrelation

Time series such as N-of-1 data often exhibit autocorrelation in which measurements taken close together are more highly correlated than measurements taken further apart in time. Autocorrelated measurements can be formed either by directly autocorrelating the outcomes or by autocorrelating the error terms. Introducing autocorrelation into the errors gives a set of equations

(2.5) $$Y_{j}=m+\sum_{k\neq 1}\delta_{k}I\left(A_{j}=k\right)+\beta t_{j}+e_{j}\;e_{j}=\rho_{e}e_{j-1}+\epsilon_{j}\;\epsilon_{j}∼N\left(0,\sigma^{2}\right)$$

where $\rho_{e}$ is the autocorrelation between $e_{j}$ and $e_{j-1}$ and $\epsilon_{j}$ is the residual error after accounting for the autocorrelation. Assuming stationarity, it follows that $e_{j}∼N\left(0,\sigma^{2}/\left(1-\rho_{e}^{2}\right)\right).$ Marginally, then

(2.6) $$Y_{j}∼N\left(m+\sum_{k\neq 1}\delta_{k}I\left(A_{j}=k\right)+\beta t_{j},\sigma^{2}/\left(1-\rho_{e}^{2}\right)\right).$$

The model can also be extended to include first-order autocorrelated outcomes as

(2.7) $$Y_{j}=m+\sum_{k\neq 1}\delta_{k}I\left(A_{j}=k\right)+\beta t_{j}+\rho_{Y}Y_{j-1}+\epsilon_{j}$$

where $\rho_{Y}$ is the autocorrelation between consecutive measurements assuming stationarity and $\epsilon_{j}$ is a random error independent of the previous outcomes. Interpretation of $\delta_{k}$ and β require care in this model because their effects are conditional on $Y_{j-1}$. In other words, $\delta_{k}$ is the treatment effect comparing two measurements taken on different treatments for which the previous outcomes were the same.

As with trend, autocorrelation for either errors or outcomes can also be added to the arm-based model in Equation 2.3.

### 2.4. Carryover

When carryover is present, one treatment is still having an effect when the next treatment starts. The carryover effect for each treatment can differ in both amount and duration. Because carryover complicates the determination of treatment effects, many trials are designed to avoid it.

A standard approach to remove carryover uses washout periods in which no treatment is given during the transition to a new treatment. Essentially, one treatment is allowed to wash out of the body before the next one begins. Because a washout period may not be practical, either because it makes trials too long or because it is not safe to withdraw treatment, N-of-1 designs may use an analytic washout in which outcomes at the beginning of crossover periods are disregarded (Kaplan et al., 2022).

Analytic washout requires untestable assumptions about the length of carryover, so it may often be necessary to model the potential carryover. But since carryover can only be measured after a treatment crossover, estimating it in a single N-of-1 trial with few crossovers is difficult. We therefore reserve discussion of modeling caryover for Section 4 when discussing the aggregation of multiple trials.

## 3. Bayesian Models

### 3.1. Using Bayes’ Rule to Form Posterior Distributions

In the Bayesian paradigm, model parameters Θ are random variables. Their probability distribution reflects current knowledge about their true values about which we have uncertainty. Bayesian inference seeks to describe our knowledge about Θ given information that the data y supply to the likelihood through the model $p\left(y|\Theta \right)$ for the data-generating process and from our prior beliefs about Θ described by the prior distribution $p\left(\Theta \right)$. The posterior distribution of $p\left(\Theta |y\right)$ describes our knowledge about Θ conditional on the known data y in terms of a probability distribution that quantifies our beliefs about the values of the parameters after (or posterior to) the data. As data accrue, the posterior continually updates to incorporate the new information. The Bayesian method therefore intrinsically incorporates the scientific learning process.

Mathematically, the prior and the likelihood combine to form the posterior through Bayes’ rule

(3.1) $$p\left(\Theta |y\right)=\frac{p\left(y|\Theta \right)p\left(\Theta \right)}{p\left(y\right)}=\frac{p\left(y|\Theta \right)p\left(\Theta \right)}{\int p\left(y|\Theta \right)p\left(\Theta \right)d\Theta }\propto p\left(y|\Theta \right)p\left(\Theta \right).$$

The denominator $p\left(y\right)$ is the marginal distribution of the data and does not depend on Θ. This greatly simplifies computing the posterior distribution. Furthermore, because Θ includes all model parameters, the posterior accounts for all uncertainty about them.

We can compute the posterior distribution for any component of Θ by integrating over the other parameters. For instance, if Θ consists of two parameters $\theta_{1}$ and $\theta_{2}$, one can compute the marginal distribution of $\theta_{1}$ by integrating over $\theta_{2}$

(3.2) $$p\left(\theta_{1}|y\right)=\frac{\int p\left(y|\theta_{1},\theta_{2}\right)p\left(\theta_{1},\theta_{2}\right)d\theta_{2}}{\int \int p\left(y|\theta_{1},\theta_{2}\right)p\left(\theta_{1},\theta_{2}\right)d\theta_{1}d\theta_{2}}.$$

Posterior inference depends on the prior and the likelihood. Because the likelihood incorporates the data-generating process, it reflects both the study design and the data model. A full Bayesian model must therefore specify design, model, and prior. The analyst needs to justify the choice of each and assess their influence via sensitivity analyses.

### 3.2. Likelihood for Single N-of-1 Trial

Using the model incorporating trend and autocorrelation given by Equation 2.6, the likelihood for a single trial can be written as

$$L=\prod_{j}p\left(Y_{j}|\text{δ},\beta ,m,\rho_{e},\sigma^{2}\right)\;=\prod_{j}\exp\left[{\left(y_{j}-M_{j}\right)}^{2}/\left(2\sigma^{2}/\left(1-\rho_{e}^{2}\right)\right)\right]/\left(\sigma /\sqrt{1-\rho_{e}^{2}}\right)$$

where $\left\{{\text{δ}}_{i}\}=\{\delta_{i_{2}},\ldots ,\delta_{i_{K}}\right\}$ is the vector of treatment effects and $M_{j}=m+\sum_{k\neq 1}\delta_{k}I\left(A_{j}=k\right)+\beta t_{j}$ is the marginal mean of $Y_{j}$.

Writing $y=\{y_{j}\}$, the posterior is then proportional to the product of the likelihood and the prior

(3.3) $$p\left(\text{δ},\beta ,m,\rho_{e},\sigma^{2}|y\right)\propto \;\;\left[\prod_{j}exp\left[{\left(y_{j}-M_{j}\right)}^{2}/\left(2\sigma^{2}/\left(1-\rho_{e}^{2}\right)\right)\right]/\left(\sigma /\sqrt{1-\rho_{e}^{2}}\right)\right]*p\left(\text{δ},\beta ,m,\rho_{e},\sigma^{2}\right).$$

To complete the model specification, we need to choose the prior.

### 3.3. Choosing a Prior Distribution

In problems with substantial information in the likelihood, the prior will have little effect on posterior inferences and it may be possible to get away with using a noninformative (flat) prior distribution. Noninformative priors can also be used as a convenience to retain the interpretive advantages of Bayesian inferences and to enable computation via simulation of posterior distributions for complex models whose likelihoods are not easily optimized. Often, it is useful to tailor the flat prior to the problem at hand by bounding it to exclude values deemed implausible by experts or by experience from the previous literature.

But when the amount of information in the data is small and the likelihood is relatively flat, the choice of prior can have a large influence on the posterior. It is then important to use all available information to construct strong priors that reflect what is known about the parameters either from historical data or from expert opinion, perhaps elicited through a structured process (Chaloner et al., 1993)(O’Hagan et al., 2006). Because different people can have different priors, they may also develop different posteriors with the same likelihoods. Often seen as a weakness, this dependence on the prior actually reflects the different choices people may make with the same information. Unfortunately, little may be known about some parameters, such as between-study variances, which require a reasonably large number of studies to be well estimated (Röver et al., 2021). In these cases, it will only be possible to construct weak priors and it will be important to try different choices to examine the sensitivity of posterior inferences to prior choices.

In practice, the priors chosen for parameters affecting the mean like m,β, and δ will rarely matter assuming that treatments are given a sufficient number of times, so it is common to choose a flat prior, often a normal distribution centered at zero with a large variance such as $N\left(0,10^{6}\right)$. An informative prior may be desired if prior knowledge of the treatments or the individual is available. For example, it may be possible to bound the potential treatment effect or the individual’s outcome levels may be approximated reasonably well.

Priors for correlation parameters like $\rho_{e}$ tend to be a bit more important. Likelihoods often have much less information about correlations and can have modes near the boundaries of ±1 (Trikalinos et al., 2014). Thus, some information in the prior may be needed to supplement the data. With enough data, though, a flat uniform prior bounded by −1 and 1 may be sufficient.

Posterior inferences tend to be most sensitive to choice of the prior for the variance, for example, $\sigma^{2}$ in this model. Using a common variance across measurements as we have makes the model more robust, but possibly at the expense of accuracy. In some problems, it may be possible to group the measurements into sets with different common variances. For instance, outcomes on one treatment may be more variable than those on another. Because the variance is always a positive number and often has a skewed distribution, symmetric prior distributions that can take positive and negative values such as the normal do not work well and one must choose a distribution with support only on the positive real line. Assuming a gamma distribution for the inverse of the variance (the precision) leads to a conjugate prior (i.e., the posterior precision is also a gamma distribution), which simplifies computation. But the parameters of the gamma distribution are not very intuitive and supposedly noninformative gamma parameters can actually be informative, so it is safer to choose a distribution whose parameters represent bounds or variation. Common choices are uniform distributions ranging between zero and an upper bound or a folded distribution such as a half-normal or half-t that only take positive values (Gelman, 2006)(Röver et al., 2021).

### 3.4. Computation via Markov Chain Monte Carlo

Markov chain Monte Carlo (MCMC) has become the primary tool for Bayesian computation because it uses an iterative numerical integration algorithm to simulate from the complete joint posterior distribution. This permits numerical calculation of any quantities of interest such as functions of parameters, predictive distributions, and the probabilities of hypotheses. It also simplifies computing for complex models by breaking them into simpler components (Gelfand & Smith, 1990).

Essentially, MCMC works by repeatedly simulating the parameters in a carefully chosen sequence such that at each step one or more of them is drawn from a known distribution conditional on the data and the current state of the other parameters. Because the sequence of draws forms a Markov chain, all of the information from the previous history of the chain is contained in the most recently sampled values of the parameters, and so the current state is the only part of the chain’s history needed to take the next sample. Crucially, it can be shown that the algorithm will converge to a stationary distribution, which is the true joint posterior distribution under mild regularity conditions that are generally satisfied for most statistical models (Roberts & Smith, 1994). Convergence may be monitored with diagnostics that check whether simulation variability is consistent with that expected from a probability distribution (Gelman, 1996). Once the Markov chain is deemed to have converged, inference is based on additional Monte Carlo samples drawn from the correct posterior distribution. Each complete pass through the algorithm results in a new draw of each parameter. The sequence of draws provides a random tour of the (high-dimensional) parameter space, visiting locations in that space with frequencies proportional to the joint posterior density. The number of additional samples should be chosen to be sufficient to obtain results with acceptable precision for making inferences. Accurate inferences about some parameters such as extreme quantiles may require more simulations than for others such as means (Gelman et al., 2013).

The output of the MCMC sequence is a full set of draws from the posterior distribution. Characteristics of any parameter, set of parameters, or function of parameters can be evaluated by empirical summaries of the drawn samples using the Monte Carlo method. For example, the median of the marginal distribution of a parameter can be estimated by the median of its sampled draws and upper and lower bounds of a posterior credible interval can be constructed from the appropriate quantiles of these same samples (e.g., a 95 % central credible interval is formed by the 2.5 and 97.5 percentiles). Because the credible interval is constructed directly from the empirical quantiles returned by the simulation, it need not be symmetric and need not assume asymptotic normality. The empirical posterior distribution also permits inferences to be made about quantities that clearly do not have normal distributions, such as the correlation or ratio between two parameters. This ability to evaluate the posterior of any parameter function is a key advantage of MCMC.

Implementing MCMC is a complex process that includes choosing efficient starting values and updating schemes, determining when the algorithm has converged and is sampling from the true posterior, and then taking a sufficiently large number of samples from the posterior distribution to limit Monte Carlo simulation error and ensure reliable inferences. The reader interested in more details should consult the extensive literature. A useful starting point is a chapter on Bayesian meta-analysis in the *Handbook of Meta-Analysis* (Schmid, Carlin, et al., 2020). Book length coverage can be found in (Brooks et al., 2011).

### 3.5. Point and Interval Estimation

Posterior inferences are often focused on marginal distributions of parameters or functions of parameters. The posterior mean, median, or mode can be used as a point estimate for a scalar parameter θ. Under a relatively flat prior, the posterior mode will be close to the maximum likelihood estimate. If the posterior is normal, the three measures are the same, but for multimodal or otherwise nonnormal posteriors, such as for a variance parameter, the mode will often be the poorest choice of centrality measure. The posterior mean will sometimes be overly influenced by heavy tails (just as the sample mean is often not robust against outlying observations). As a result, the posterior median will often be the best and safest point estimate and is relatively easy to compute using MCMC.

The posterior distribution allows us to make direct statements about not just its median, but any quantile or interval. For instance, a $100\times \left(1-\alpha \right)$% credible interval for θ is an interval $\left(q_{L},q_{U}\right)$ such that $P\left(q_{L}<\theta <q_{U}|y\right)=1-\alpha$. Such an interval is easy to compute from MCMC simulations, and has a direct interpretation as an interval within which θ lies with probability $\left(1-\alpha \right)$. Most commonly, a symmetric interval is chosen to exclude α/2 of the probability on each side, although for positively valued parameters like variances, one-tailed intervals may be preferred. Unlike a frequentist confidence interval, the credible interval provides a direct probabilistic interpretation of the specific numerical credible interval. If little prior information is available about model parameters, a credible interval and a confidence interval may be numerically similar, though.

Note also that p values, which are probabilities about the likelihood of specific null hypotheses, do not have a role in Bayesian inference. Instead, Bayesians report the posterior probability of hypotheses of interest. This does not preclude testing of hypotheses that may be needed for confirmatory testing, though, because one can always formulate the test in terms of a required posterior probability about a particular hypothesis, such as that the treatment is better than the control by a certain amount with at least a certain prespecified probability.

### 3.6. Prediction

Making predictions is often the primary motivation for doing a statistical analysis. Predictions are useful with N-of-1 trials in several situations. One might want to predict the results of a new trial or one might wish to predict the eventual result of an ongoing trial. In both situations, the Bayesian approach is well suited to making predictions through the use of the predictive distribution.

Let $p\left(\Theta \right)$ represent the current information about the model parameters. This may be based on previous data y in which case we could use a posterior distribution $p\left(\Theta |y\right)$. Because the posterior distribution conditional on the previous data y can be thought of as the prior distribution before collecting the new data $y^{new}$, we can work with either notation. Recognizing that the prior distribution is often based on past information, we will simply condition on the past history noted as H.

The posterior predictive distribution for $y^{new}$ is found by averaging the conditional predictive distribution $p\left(y^{new}|\Theta \right)$ with respect to the prior distribution $p\left(\Theta \right)$. The predictive distribution for new data $y^{new}$ may then be written

$$p\left(y^{new}|H\right)=\int p\left(y^{new}|\Theta \right)p\left(\Theta |H\right)d\Theta .$$

The predictive distribution can be estimated by simulating Θ many times from $p\left(\Theta |H\right)$ and then drawing from $p\left(y^{new}|\Theta \right)$ for each simulated Θ. When Θ is being drawn from a posterior distribution, the MCMC samples of Θ may be used as the draws from $p\left(\Theta |H\right)$. The predictive distribution for a new treatment effect $\theta^{new}$ can likewise be written

$$p\left(\theta^{new}|H\right)=\int p\left(\theta^{new}|\Theta \right)p\left(\Theta |H\right)d\Theta .$$

Because the predictive distribution captures the uncertainty in both the true value of the model parameters expressed by $p\left(\Theta |H\right)$ as well as the uncertainty of the individual outcome drawn from the model in $p\left(y^{new}|\Theta \right)$, credible intervals for predictions are often much wider than those for model parameters. This can lead to conclusions that one treatment may be better than another on average, but not necessarily in individual trials.

For example, consider a disease condition with binomial outcomes for which the posterior probability that a treatment is successful is highly concentrated near 0.8. One would call this a useful treatment, especially if the alternative always failed. And yet, the treatment will fail in one out of every five future patients that are treated. One can apply similar logic to the likelihood that an N-of-1 trial will succeed if rerun, particularly if the new attempt is much shorter than the original. In general, one can predict the likelihood of any future event E by substituting E for $y^{new}$ above.

### 3.7. Missing Data

Because N-of-1 data are often collected and recorded by participants themselves in the course of their daily lives, missing values are common (Marcus et al., 2022)(Kaplan et al., 2022), Proper analysis with missing data requires knowledge of the missing data mechanism and whether the reason that the data are missing is related to measured or unmeasured variables. It is therefore important to collect information on why data are missing.

If the missing information is only related to variables included in the model, that is, if they are missing at random, then missing measurements may be imputed based on the model. Multiple imputation is straightforward with Bayesian models because the missing values may be treated as model parameters. MCMC then simulates from their correct posterior distribution (Lunn et al., 2013). For single trials, models with trend and autocorrelation can therefore ignore the missing values if it can be assumed that the reason they are missing is independent of external factors.

If the data are missing-not-at-random, then more complex models that account for the cause of the missing data would need to be developed. For example, if a participant becomes ill and cannot enter data and if the outcome is health-related, then analyzing the available data or imputing from a model based on the available data may lead to a biased result. If some information is available about outcomes during illness periods, that could be used to build an imputation model. The analysis of the PRODUCE data in Section 5 provides an example of constructing a missing-not-at-random imputation model in which the imputation model depends on the time that the participant dropped out of the study.

### 3.8. Model Checking: Posterior Predictive Checks

External validation in which a model constructed from one data set is applied to the data from a new data set with the new predictions compared to the new outcomes is the gold standard for model validation. New data sets are hard to find, though, so we often must make do with with internal validation using the data available. With any internal validation method, a good model should be able to replicate the observed data. For a Bayesian, this entails simulating data from the posterior predictive distribution and checking how well it compares to the observed data. The observed data should look plausible under the posterior predictive distribution. To carry out a posterior predictive check, we first simulate outcomes $y^{rep}$ from the posterior predictive distribution by drawing Θ from its posterior $p\left(\Theta |y\right)$, and then drawing $y^{rep}|\Theta$. We then evaluate the test statistic $T\left(y,\Theta \right)$ at both $y^{rep}$ and y and compare them. A posterior predictive p value, $P\left(T\left(y^{rep},\Theta \right)\geq T\left(y,\Theta \right)|y\right)$, is calculated as the proportion of the number of samples l=1,2,…,L such that $T\left(y^{rep},\Theta^{l}\right)\geq T\left(y,\Theta^{l}\right)$. Note that this expression conditions on both y and Θ, so test quantities can also be functions of the unknown parameters Θ (Gelman et al., 2013).

In a typical N-of-1 trial, one is not interested in making predictions about other individuals, although one might be interested in predicting the future course of a particular individual. Nevertheless, rarely would one have enough information to develop such a model and so posterior predictive checks would not be indicated.

When aggregating across multiple individuals as outlined in the next section, though, it might be important to model the population in such a way that a prediction could be made for a new individual, or it might be important to transport the model to a new population. In such a case, posterior predictive model checks may be needed. We do not pursue them further in this article, however, as the application to follow is not focused on making predictions on new individuals.

## 4. Aggregating Data Across Multiple Individuals

When different individuals carry out N-of-1 trials with a similar design, it may be possible to aggregate them with a multilevel model to learn about the group and to learn more about each individual. In essence, the aggregated trials form a meta-analysis of the individual trials. The first level of the multilevel model applies to the individual and the second applies to the group.

The first level can be written using the same notation as for the individual models in Section 2 except that we add a subscript i to reference the individual. For the contrast-based linear trend model with autocorrelation

(4.1) $$Y_{ij}=\mu_{i}+\sum_{k\neq 1}\delta_{ik}I\left(A_{ij}=k\right)+\beta_{i}t_{ij}+e_{ij}$$

(4.2) $$e_{ij}=\rho_{e_{i}}e_{ij-1}+\epsilon_{ij}$$

(4.3) $$\epsilon_{ij}∼N\left(0,\sigma_{i}^{2}\right),$$

each individual has distinct parameters $\mu_{i},\;\delta_{ik},\;\beta_{i},\;\rho_{e_{i}}$, and $\sigma_{i}^{2}$. Analogous formulas apply if the treatments are expressed in an arm-based format.

### 4.1. Common, Fixed, and Random Trial-Level Parameters

Analysts typically handle these individual study-level parameters in one of three ways. First, one can assume they are the same across studies, reducing them to one common parameter, for example, $\delta_{ik}=\delta_{k}$ for all i. This is a strong assumption that simplifies computations but requires justification (Schmid, Stijnen, et al., 2020a). Alternatively, the first-level parameters may be related to each other by assuming they come from a common distribution. Doing so treats each individual parameter as a random draw from the common distribution and so they are called random parameters (or random effects). Under the random effects model, both the individual-level parameters and their common mean and variance, for example, $\delta_{k}$ and $\sigma_{\delta }^{2}$, respectively, (the second-level parameters) have posterior distributions. The posterior distributions of the individual (first) level parameters, for example, $\delta_{ik}$ turn out to be mixtures of their individual likelihoods and their common distribution. Essentially, by treating the parameters as related, we estimate their posteriors to be more alike than they would be if they were left unrelated. A third approach leaves the first-level parameters unrelated, in which case they are called fixed parameters (effects) and their posterior distributions are left unaffected by the rest of the model. Using fixed parameters precludes drawing any inferences beyond the trials modeled and thus are only appropriate if the focus is entirely on drawing inferences for those individuals studied. If one wants to draw inferences about the population from which these individuals are drawn, random effects should be used.

Random treatment effects $\delta_{ik}$ and trend slopes $\beta_{i}$ are typically assumed to come from common normal distributions

(4.4) $$\delta_{ik}∼N\left(d_{k},\sigma_{\delta }^{2}\right);\;\;k=2,\ldots ,K_{i}$$

where $K_{i}$ is the number of treatments in trial i and

(4.5) $$\beta_{i}∼N\left(b,\sigma_{\beta }^{2}\right).$$

When the outcomes in each trial are normally distributed given the first-level parameters and the first-level parameters are themselves drawn from a normal distribution, the posterior distributions of the first-level parameters are normal mixtures of these two distributions with mixing weights that are proportional to their relative precision, or inverse variances (Schmid, Carlin, et al., 2020). The resulting posterior is shrunk toward or close to the common distribution if the individual-level data are imprecise or if the individual effects are homogeneous. On the other hand, if an individual’s parameters are well-estimated or if the set of individual effects is heterogeneous, then the individual’s posterior estimates will not be shrunk toward the common group distribution.

We can also treat the intercepts $\mu_{i}$ as random or fixed parameters. A common intercept is usually considered unreasonable because individuals would be expected to have heterogeneous outcomes as a result of underlying individual characteristics. Treating the intercepts as random

(4.6) $$\mu_{i}∼N\left(m,\sigma_{\mu }^{2}\right)$$

will result in their influencing the estimation of other parameters in the model. In particular, individual treatment effects are informed by differences in the mean outcome levels between trials (White et al., 2019). Because of this shrinkage, the meta-analysis literature has a long-standing controversy over whether to treat the intercepts as fixed or random (Senn, 2000). Particularly for properly designed and conducted randomized trials that provide causal estimates of treatment effects, many consider the use of random intercepts that can influence those treatment effects to be inappropriate (Dias & Ades, 2016). Instead, they argue for deriving inferences conditional on their fixed values. Essentially, this treats the intercepts as nuisance parameters that can be ignored in drawing inferences about the other parameters. However, treating them as fixed precludes drawing inferences about the intercepts from new studies and so limits prediction.

If the intercepts are treated as random, then the model can also be extended to allow correlation between the intercepts and the treatment effects (Stijnen et al., 2020). As the intercept can be considered to be the average outcome under the reference treatment and the treatment effects are contrasts between each treatment and the reference, it makes sense that these would be related. Let us write $\Sigma_{\mu \delta }$ for the covariance matrix for ${\left[\mu_{i},\delta_{i2},\ldots ,\delta_{iK_{i}}\right]}^{T}.$ Because the $\delta_{ik}$ must be consistent within a trial, the treatment effect between levels k and k' must equal the difference $\delta_{ik}-\delta_{ik'}$. Under the assumption of a constant treatment variance, all treatment contrasts must have the same variance. This implies that the correlation between the $\delta_{ik}$ must then be 0.5 (Higgins & Whitehead, 1996). Assume that the correlation between $\mu_{i}$ and each $\delta_{ik}$ is identically $\rho_{\mu \delta }$. Then

(4.7) $$\Sigma_{\mu \delta }=\left(\begin{array}{cc} \sigma_{\mu }^{2} & \rho_{\mu \delta }\sigma_{\mu }\sigma_{\delta }1_{K_{i-1}}^{⊺}\\ \rho_{\mu \delta }\sigma_{\mu }\sigma_{\delta }1_{K_{i-1}}^{⊺} & \sigma_{\delta }^{2}P_{K_{i}}\left(0.5\right) \end{array}\right)$$

where $1_{K_{i}-1}$ is a $K_{i}-1$ length vector of ones and $P_{K_{i}}\left(x\right)$ is a $K_{i}\times K_{i}$ matrix with all diagonal elements equal to one and all off-diagonal elements equal to 0.5.

The within-individual correlations $\rho_{e_{i}}$ are slightly more complicated to model because they are typically skewed and bounded. Thus, they cannot be treated as normally distributed. To avoid this issue, one can assume common or fixed correlations, although this carries the limitations discussed above. Random effects formulations commonly work by applying a transformation that normalizes their distributions. It is common to use the inverse hyperbolic tangent transformation $z_{e_{i}}=\frac{1}{2}ln\left(\frac{1+\rho_{e_{i}}}{1-\rho_{e_{i}}}\right)$ and assume that

(4.8) $$z_{e_{i}}∼N\left(z_{e},\sigma_{z_{e}}^{2}\right).$$

Using MCMC, one can easily recover the posterior distribution of the $\rho_{e_{i}}$ from the posterior samples of $z_{e_{i}}$ by applying the hyperbolic transformation $\rho_{e_{i}}=exp\left(2z_{e_{i}}-1\right)/\left(1+exp\left(2z_{e_{i}}\right)\right)$.

Finally, it is easiest to treat the variances $\sigma_{i}^{2}$ as fixed parameters unless one is interested in modeling them. Alternatively, a common residual variance is often assumed so that $\sigma_{i}^{2}=\sigma^{2}$ for all i.

### 4.2. Multilevel Models and Hyperparameters

Parameters of the common distribution from which trial-level random effects are drawn are called hyperparameters because they are parameters of parameters. Combining Equations 4.1–4.8 gives a multilevel model where $\left\{d_{k}\right\}$, $b,\;m,\;z_{e},\;\rho_{\mu }\delta ,\;\sigma_{\delta }^{2},\;\sigma_{\beta }^{2},\;\sigma_{\mu }^{2}$ and $\sigma_{z_{e}}^{2}$ are the hyperparameters. The full set of model parameters Θ includes the hyperparameters and the study-level parameters $\sigma_{i}^{2}$, $\left\{\beta_{i}\right\}$, $\left\{\mu_{i}\right\}$, $\left\{z_{e_{i}}\right\}$ (or $\left\{\rho_{e_{i}}\right\}$), and $\left\{{\text{δ}}_{i}\right\}$. Assuming the trials independent, the likelihood L for the data $y=\{y_{ij}\}$ can be written

$$L=\prod_{i}p\left(y_{i}|\{{\text{δ}}_{i}\},\beta_{i},\mu_{i},z_{e_{i}},\sigma_{i}^{2}\right)p\left(\{{\text{δ}}_{i}\},\mu_{i}|\{d_{k}\},m,\sigma_{\delta }^{2},\sigma_{\mu }^{2},\rho_{\mu \delta }\right)p\left(\beta_{i}|b,\sigma_{\beta }^{2}\right)p\left(\{z_{e_{i}}|,z_{e},\sigma_{z_{e}^{2}}\right).$$

The posterior $p\left(\Theta |y\right)$ is then the product of the prior $p\left(\{d_{k}\},b,m,z_{e},\sigma_{\delta }^{2},\sigma_{\beta }^{2},\sigma_{\mu }^{2},\sigma_{z_{e}}^{2},\sigma_{i}^{2}\right)$ and this likelihood. We can compute the marginal posterior distribution for any component of Θ by integrating over the other parameters. This is straightforward with MCMC because we simply use the simulations of the parameters of interest.

When aggregating N-of-1 trials, both first- and second-level parameters are important because we want to draw inferences about both the individuals and the population. The posterior distributions of the individual parameters also inform about the true heterogeneity among individuals (as distinguished from sampling variation that occurs when the number of measurements taken from an individual is too small). As discussed above, the posterior estimates of individuals are affected by data from others through their common second-level distribution. We say that the individuals borrow strength from each other. Although it might seem strange that a better estimate of the effect of an individual may be gained by using data from other individuals, this phenomenon, called shrinkage, is well known in statistical theory (Efron & Morris, 1973)(James & Stein, 1961). Intuitively, if the individual effects come from a common distribution or are exchangeable in statistical terminology, then we can gain more information about each one by using information from the others (Draper et al., 1993). This phenomenon describes the standard way we learn about new things by using what we know about similar things.

### 4.3. Modeling Within and Between-Individual Heterogeneity

When individual effects exhibit heterogeneity, it may be worthwhile to try to characterize the between-individual heterogeneity in terms of baseline characteristics that apply to subgroups of individuals, for example, men and women or older and younger individuals. Variables representing these characteristics can be included as regression terms $x_{jk}$ in the expression for the mean of the treatment effects in the second-level model 4.4 as $d_{k}=d_{0k}+\sum_{j=1}^{J}d_{jk}x_{jk}$. Heterogeneity among the intercepts $\mu_{i}$ or the trends $\beta_{i}$ may also be modeled by reformulating their means as regressions. Such covariates vary between but not within individuals. In addition to varying between individuals, outcomes may vary within individuals as a function of covariates $z_{l}$ too. These may be introduced into Equation 4.1 as

(4.9) $$y_{ij}=\mu_{i}+\sum_{k\neq 1}\delta_{ik}I\left(A_{ij}=k\right)+\beta_{i}t_{ij}+\sum_{l=1}^{L}\gamma_{li}z_{l_{ij}}+e_{ij}$$

### 4.4. Models for Carryover

Carryover is difficult to estimate in a single individual with only a few crossovers. With data from multiple individuals, however, the number of treatment crossovers is much larger and carryover is estimable for any treatment sequence that is repeated often enough (assuming of course some pattern in carryover such as that it is stable across time and across different individuals). Considering pairs of treatments, we can estimate crossover from one to the other in either order so the total number of possible crossover parameters for K treatments is 2×K2.

One might also consider more complex ordering effects in which the effect of a treatment depends on more than the previous treatment. Often, scientific knowledge informs which crossovers to include in a model. For instance, switching from a placebo should not induce a crossover effect. In designs with repeating sequences such as ABAB designs, the crossover effect can become confounded with the sequence effect.

Carryover can be also incorporated into models by introducing covariates that describe the potential carryover effect. For instance, carryover from a pharmacological treatment that continues to act after it is discontinued can be captured by using an indicator variable that is *on* when the carryover may be present and is *off* otherwise.

If the drug loses potency over time, then the modeled carryover effect can be more complex, perhaps taking a positive fraction to reflect the treatment’s decline in potency. For example, carryover from a treatment period into the following placebo period for a drug with half-life of one time unit may be modeled by including a variable $z_{1_{ij}}$ in Equation 4.9 such that $z_{1_{ij}}=2^{-\left(t_{ij}-t_{ij}^{*}\right)}$ where $t_{ij}^{*}$ is the time when the crossover occurred. In this case, $\gamma_{1i}=\delta_{ik}$, so the total effect at $t_{ij}$ is $\delta_{ik}\left(1+z_{1_{ij}}\right)$

### 4.5. Missing Data

Handling missing values becomes more complicated when aggregating trials because the causes of missing values often vary from individual to individual. If these can be captured in covariates that can be modeled, it is possible to multiply impute values from a model conditional on these covariates, both within and between individuals. Missing values can again be treated as model parameters and MCMC will correctly update them from the regression model. This approach may become impractical as the number of individuals becomes large, though, because the number of parameters to simulate will grow rapidly. With data missing not-at-random, models must incorporate missing data mechanisms that vary across individuals. The analysis of the PRODUCE data in the next section provides an example of constructing a missing-not-at-random imputation model in which the imputation model depends on the time that the participant dropped out of the study.

## 5. Example: PRODUCE Study

To illustrate these techniques, we turn to a set of N-of-1 trials in the PRODUCE study that we helped to design and analyze (Kaplan et al., 2019)(Kaplan et al., 2022). PRODUCE compared usual diet (UD) to two modified diets, the Specific Carbohydrate Diet (SCD) and a less restrictive modified SCD (MSCD), for treatment of pediatric noninflammatory bowel disease (IBD). Children had either Crohn’s disease (CD), ulcerative colitis (UC), or indeterminate colitis (IC). Following a 2-week baseline period of UD, participants were randomized to either SCD or MSCD. Each diet was maintained for 8 weeks at which point participants crossed over to the other experimental diet for 8 more weeks. Participants then repeated this 16-week sequence in the same order so that they followed either an ABAB or a BABA randomization sequence. Sequences were repeated to avoid 16 consecutive weeks on the stricter SCD diet, which patients who helped to design the study thought might lead to increased dropout.

Participants were allowed to cross over to the next treatment at any time before 8 weeks and were also allowed to discontinue the study at any time. Following completion, they received graphical and textual information about their performance, which included the probability that SCD and MSCD improved outcomes compared to UD and also compared to each other. A variety of patient-reported outcomes including stool frequency, stool consistency, pain interference, IBD symptoms, and self-reported disease activity, as well as a laboratory measure of intestinal inflammation via fecal calprotectin were collected and analyzed. Here, we illustrate the analysis of the weekly $\text{PROMIS} ^{\text{\tiny{\textregistered}}}$ Pain Interference Scale, which is reported as a T-score measure (standardized mean of 50 and standard deviation of 10) and has a range from 38 to 78 if reported by parents and 34 to 78 if reported by children. A clinically important change is defined as a 3-point change in the scale so that an increase of at least 3 points indicates improvement and a decrease of at least 3 points indicates worsening.

Among 54 randomized participants, 21 completed the full four-period sequence, 9 completed the study early after a single crossover (two periods), and 24 withdrew during the first or second period before completing both diets. To avoid issues with potential carryover, we did not analyze the first weekly measurement in any of the four experimental diet periods, so each period had a maximum of 7 measurements.

### 5.1. Analysis of Individual Trials

We analyzed the pain score as a continuous variable for each individual with an arm-based model that included autocorrelation, normally distributed errors, but no trend. Missing observations were imputed as parameters in the Bayesian model under the assumption that they were missing at random.

We chose noninformative prior distributions for the model parameters using a Uniform(34,78) for the treatment means $\alpha_{k}$ when reported by the child and U(38,78) when reported by the parent to reflect the range of the pain outcome scale, a U(−1,1) for the correlation $\rho_{e}$ and a U(0,1000) for the standard deviation σ.

**Figure 1.**
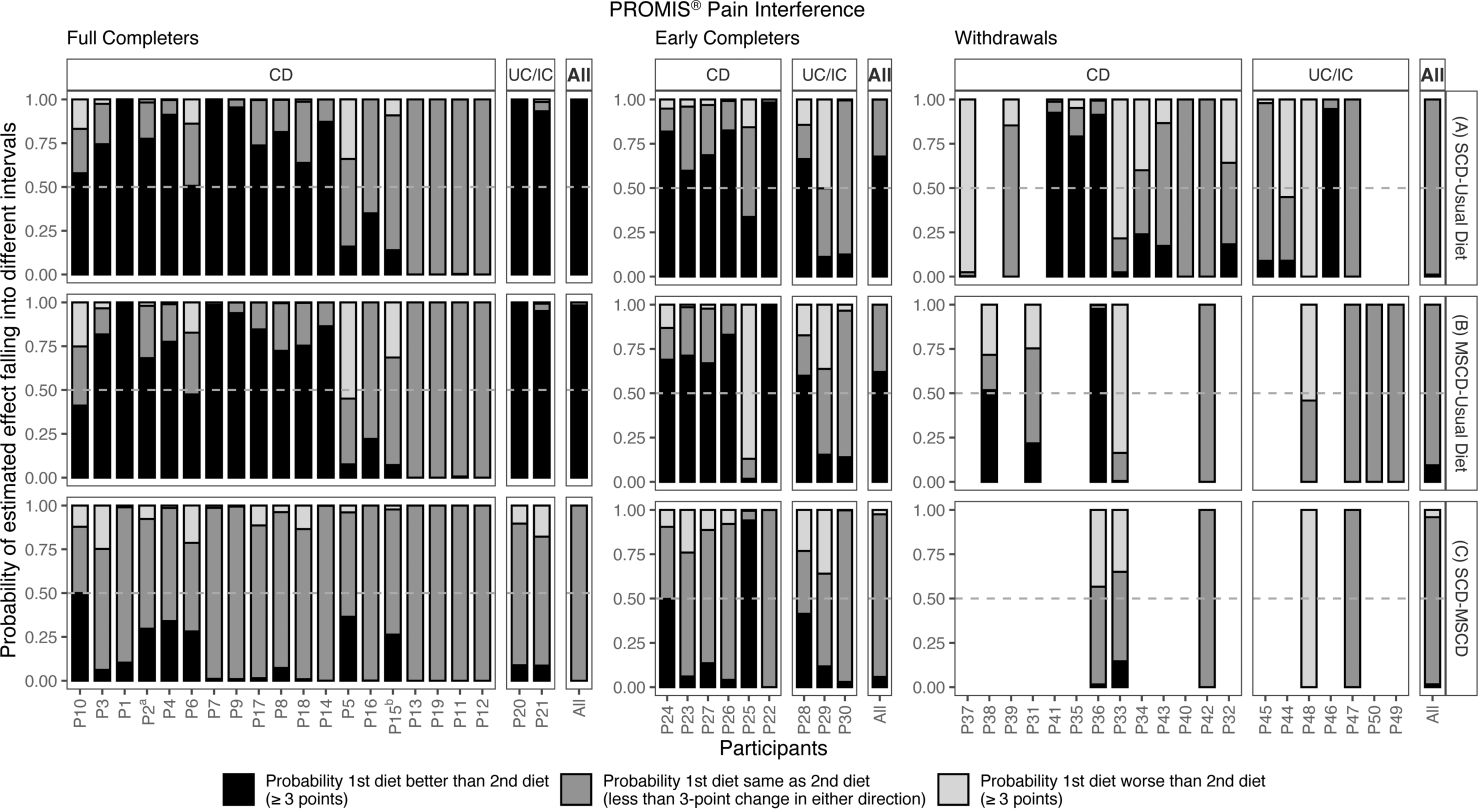
Posterior probability of symptomatic improvement in pain interference in individual N-of-1 trials for full completers, early completers, and withdrawals for three diet comparisons: (A) SCD versus Baseline/Usual Diet, (B) MSCD versus Baseline/Usual Diet, and (C) SCD versus MSCD. Within each diet comparison, individual trial probabilities are ordered by disease type and by extent of baseline symptoms (more to less). For withdrawals, participants with measurements only on baseline diet are not included in the figure. Note: a indicates that a child response rather than a parent response was used in analysis, b indicates that the participant was randomized to begin with SCD, but began with MSCD. *Note*. CD: Crohn’s disease; UC: ulcerative colitis; IC indeterminate colitis; UD usual diet; SCD specific carbohydrate diet; MSCD modified specific carbohydrate diet

**Figure 2.**
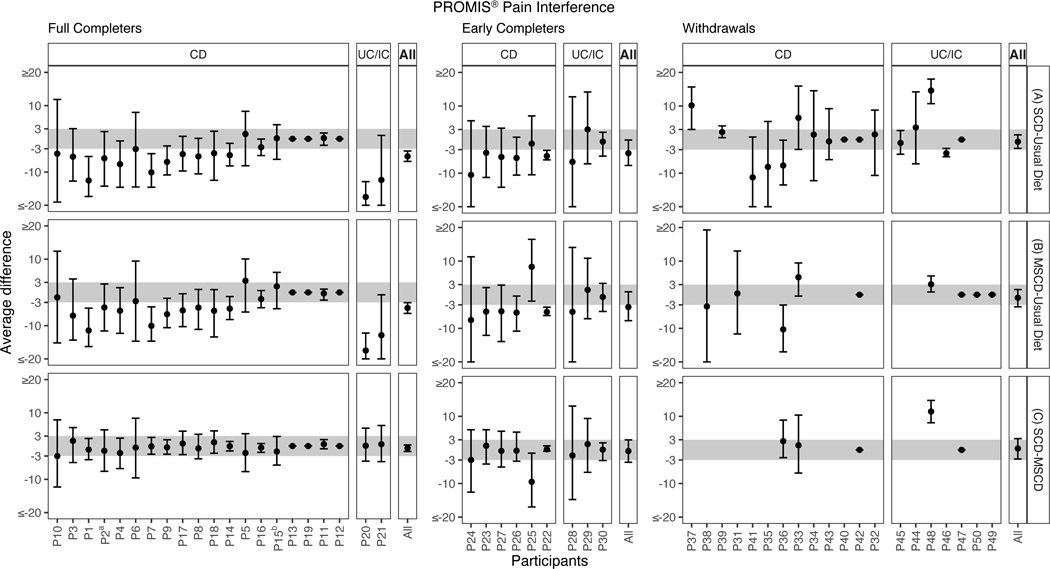
Posterior median and 95% credible interval of the difference of symptomatic improvement in pain interference in individual N-of-1 trials for full completers, early completers, and withdrawals for three diet comparisons: (A) SCD versus Baseline/Usual Diet, (B) MSCD versus Baseline/Usual Diet, and (C) SCD versus MSCD. Within each diet comparison, individual trial probabilities are ordered by disease type and by extent of baseline symptoms (more to less). For withdrawals, participants with measurements only on baseline diet are not included in the figure. Note: a indicates that a child response rather than a parent response was used in analysis, b indicates that the participant was randomized to begin with SCD, but began with MSCD. *Note.* CD: Crohn’s disease; UC: ulcerative colitis; IC indeterminate colitis; UD usual diet; SCD specific carbohydrate diet; MSCD modified specific carbohydrate diet

Individual patient posterior probabilities for each diet comparison including SCD vs. UD (Panel A), MSCD vs. UD (Panel B), and SCD vs. MSCD (Panel C) are shown in Figure 1. The corresponding median posterior treatment difference and 95% CrI are shown in Figure 2. The probability of improvement on SCD vs. UD varied by individual. Twelve of the full completers, 5 of the early completers, and 4 of the withdrawals were classified as responders, having a >50% probability of clinically meaningful improvement of 3 points and a <10% probability of worsened pain interference on SCD compared to UD. Similar heterogeneity was seen in the individual probabilities of improvement in pain interference on the MSCD versus UD. Twelve of the full completers, 4 of the early completers, and 1 of the withdrawals had a >50% probability of clinically meaningful improvement and a <10% probability of worsened pain interference on the MSCD as compared to the UD. Most participants showed minimal differences between the SCD and MSCD.

### 5.2. Analysis of Aggregated Trials

We also separately meta-analyzed each of the three sets of participants (full completers, early completers and withdrawals) with multilevel models using fixed intercepts, a common autocorrelation $\rho_{e}$ and a common residual variance $\sigma^{2}$ to obtain an average effect size. The use of fixed intercepts implicitly adjusts for the two factors (clinical site and disease condition) on which participants were stratified in randomization. Because results for the individual analyses are similar both with and without imputation, we ignore the missing values when combining participants in each set, analyzing only the observed outcome data in the meta-analysis for computational efficiency.

Prior distributions were again chosen to be noninformative with a U(34,78) distribution for $\mu_{i}$, U(−44,44) for $d_{k}$, U(−1,1) for $\rho_{e}$, and U(0,1000) distributions for σ and $\sigma_{\delta }$.

Posterior medians, 95% credible intervals, and posterior probabilities of benefit and harm are shown in Figures 1 and 2 under the heading *All*. Overall, the SCD and the MSCD were almost certainly more effective than UD for full completers, had a greater than 50% chance of being more effective for early completers, but were not better for withdrawals. No differences were found between SCD and MSCD in any of the three groups.

Finally, we combined the full completers, early completers, and withdrawals together to derive an average effect across all participants, and multiply imputing missing values to form five complete data sets. Because the patient results differed so much by their stage of completion, we imputed values in each set separately based on the modeling results from each group alone. Both intermittent missing measurements and missing measurements due to dropout were imputed to ensure that all participants had at least one weekly measurement on UD and at least six in each SCD or MSCD period. Estimates from the five imputations were then combined using Rubin’s rules for multiple imputation (Rubin, 2004). On average, pain was decreased by −3.0 (95% CrI −4.2, −1.8) points on SCD compared to UD. The posterior probability was 0.48 that SCD was better than UD, 0.52 that they were no different, and <0.01 that it was worse. Very similar results applied to MSCD.

To explore heterogeneity of treatment effects by different clinical characteristics, we included a term in the second-level model as in Equation 4.9. Table 1 shows results for girls and boys. Girls had larger improvements on both diets than boys with a 4.8 compared to 1.6 point improvement for SCD and a 4.5 compared to 1.7 point improvement for MSCD. The probability that improvement was more for girls than boys was 0.99 for SCD and 0.97 for MSCD.

Analysis of the individual and aggregated trials shows that both diets reduced pain on average for this group of children, but that some individuals had no benefit. Benefit was much more likely among those who finished the study and least likely among those who withdrew early. Practically, this finding may suggest that diet therapy may be worth trying but that some individuals may not tolerate or improve from it. Girls may also benefit more than boys, perhaps because they were more adherent or for some unknown biological reason, although since this comparison was exploratory, all conclusions are purely speculative.

## 6. Extending Models to Other Data Structures

Thus far, we have considered only continuous outcomes that can be modeled with normal error distributions and sets of trials in which each individual receives the same set of treatments. Here, we outline some possible approaches for handling discrete outcomes and discuss how network meta-analysis methods could be used for data in which individuals receive only a subset of potential treatments. Such data structures occur in many studies. For instance, the I-STOP-AFib study tested whether certain activities might trigger episodes of atrial fibrillation (AF). The episodes were treated as binary outcomes (Marcus et al., 2022). In the PREEMPT study, individuals with chronic pain were allowed to design their own trials and chose a wide variety of treatment pairs (Barr et al., 2015).

### 6.1. Discrete Outcomes

For discrete outcomes, such as categorical and count outcomes, we can adopt the generalized linear model in which

(6.1) $$g\left[E\left(Y_{j}\right)\right]=m+\sum_{k\neq 1}\delta_{k}I\left(A_{j}=k\right)$$

for the contrast-based treatment effects model and

(6.2) $$g\left[E\left(Y_{j}\right)\right]=\sum_{k}m_{k}I\left(A_{j}=k\right).$$

for the arm-based treatment effects model. Notation is similar to the previous linear models except that $g\left(\cdot \right)$ is a link function relating the expected value of the outcome to the linear predictor. Independent binary outcomes $Y_{j}$ take Bernoulli distributions with probabilities $p_{j},\;E\left(Y_{j}\right)=p_{j}$ and have a link function that is generally taken to be a logit or probit function. For count outcomes, $Y_j \sim \textnormal{Poisson}(\lambda_j)$ where $\lambda_{j}$ is the rate of events at time t. The link function is then $log\left(\lambda_{j}\right)$. For categorical outcomes, $Y_{j}$ can take on one of M discrete values m=1,…,M with probabilities $p_{jm}$ such that $\sum p_{jm}=1$. This describes a multinomial distribution. A variety of different models can be constructed to relate these probabilities to each other and to the linear predictors. When the categories are unordered it is common to use a baseline category logit model in which the linear predictor is set equal to $p_{m}/p_{0}$ for m=2,…,M. Ordered categories can take several different forms, of which the most common is the cumulative logit with the linear predictor is set equal to $\sum_{m>m_{0}}p_{m}/\sum_{m\leq m_{0}}p_{m}$ for $m_{0}=1,\ldots ,M-1$. The linear model for continuous outcomes can also be written as a generalized linear model where $g\left(\cdot \right)$ is the identity link function and the data follow a normal distribution with variance $\sigma^{2}$.

Models for discrete outcomes are easily extended to incorporate trend, but autocorrelation is a bit trickier because of the lack of an error term. Instead, one needs to express the discrete outcome in terms of a latent continuous variable on which scale the autocorrelation can be modeled (Zeger, 1988).

### 6.2. Networks of Trials

Individual trials in a collection of N-of-1 trials may not share the same treatment sets. For example, the PREEMPT trial allowed participants to choose the two treatments they wanted to compare. As a result, the 98 trials had many different treatment pairs, many of which were unique. The different treatment comparisons then form a network in which the treatments are nodes. Two nodes are connected by trials that compare their treatments. This type of structure is the same as that in a network meta-analysis and methods of network meta-analysis can be applied (Dias et al., 2018). Analytically, this poses no real difficulties as the multilevel models are similar except that only a small number of treatment effects will be observed in any one trial and so a given treatment effect $\delta_{ik}$ may only contribute to a small proportion of trials.

To be able to estimate each treatment comparison, however, one must make the strong transitivity assumption that the treatments missing in any given trial are missing at random (Salanti, 2012). This has several implications, one of which is that every missing treatment pair would have had the same expected effect in the trials for which it was missing as it had in the trials in which it was observed. Because the choice to test some treatments and not others is often related to the outcomes expected (e.g., one would not test a treatment that one knew did not work or was not practical), this assumption of transitivity is probably even more suspect in collections of N-of-1 trials than in the collections of randomized controlled trials that often form a network meta-analysis. Thus, it needs to be used with extreme care.

## 7. Practical Issues

**Figure 3.**
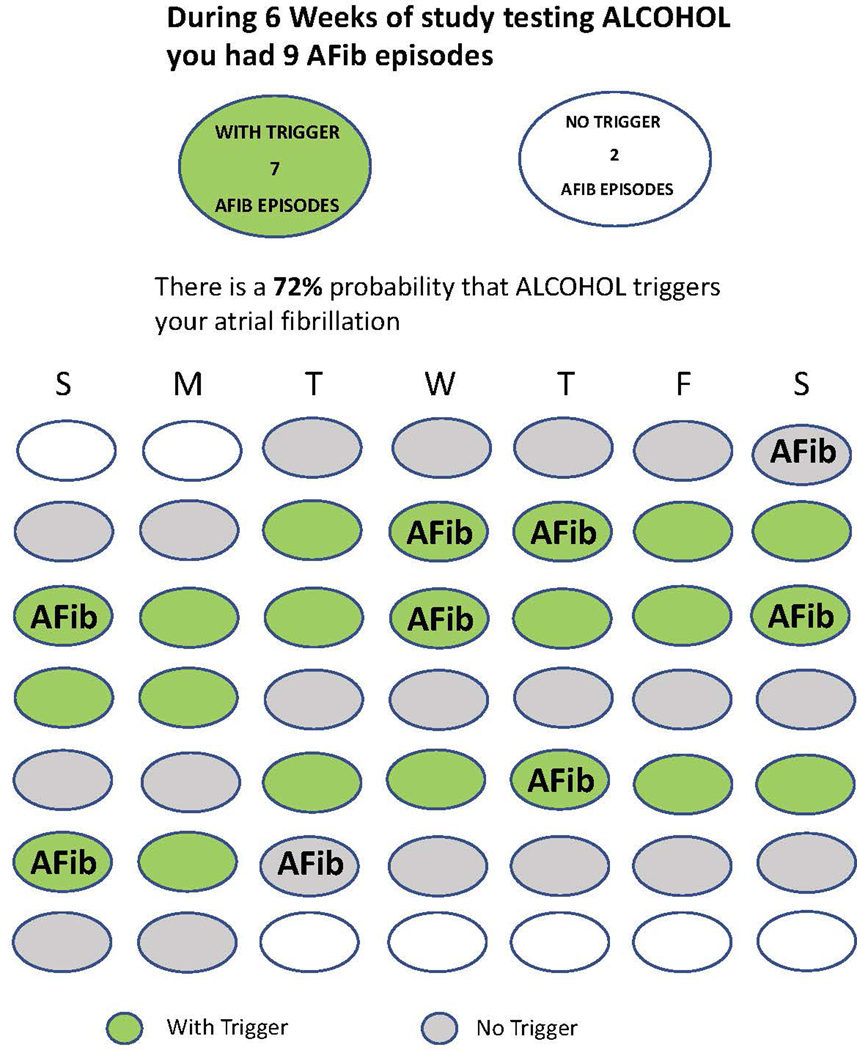
Screenshot of report given to participants in the I-STOP-AFib study following a 6-week N-of-1 trial testing a potential trigger of atrial fibrillation.

Because N-of-1 trials are focused on facilitating decision-making by and for individuals, it is important that results be made understandable to those individuals or their agents. Bayesian models provide probabilistic answers that reflect uncertainty in knowledge about key parameters such as intervention effects. Discussions with users either when planning trials or in debriefing after trials have emphasized/revealed that many people have trouble understanding probabilities and uncertainty (Kaplan et al., 2019)(Whitney et al., 2018). Our experience has been that most people are comfortable with averages, but do not appreciate that averages alone without uncertainty estimates lead to poor decisions. Sometimes, the best option may just be to provide the data. For example, in the I-STOP-AFib study testing whether certain activities might trigger episodes of atrial fibrillation (AF), participants were followed for 6 weeks, 3 on a potential trigger and 3 off. After 6 weeks, they were given a graph like that in Figure 3, arranging their treatment periods in a calendar form with days of AF episodes noted and provided the posterior probability that events were more likely when using the trigger than when not (Marcus et al., 2022).

People also interpret probabilities themselves quite differently. Some will choose an option that has probability greater than 0.5; others require a greater degree of certainty (Zucker et al., 2006). Of course, the relative costs of different options also play a role in the decision, so personalized trials should incorporate formal decision modeling to make such choices transparent. In general, more research is needed into how to make the patient experience more educational and less intimidating.

Successful implementations of N-of-1 trials using mobile applications are becoming more common (Daskalova et al., 2016)(Kaplan et al., 2022)(Kravitz et al., 2018)(Kravitz et al., 2020)(Marcus et al., 2022). The display in Figure 3 was generated on a mobile phone using an application developed specially for the I-STOP-AFib study. The mobile app carries out many of the functions such as randomization, data entry, participant followup and data analysis provided by humans at great cost in standard clinical trials. It provides users with text reminders and motivational messages to keep them involved and committed. It also reduces the costs of the trial by automating many procedures that usually require considerable staff effort.

In discussions with researchers, participants have offered many reasons why they have chosen and liked N-of-1 trials (n.d.-c)(Whitney et al., 2018). They like the personalized learning approach in which the study is tailored directly to their needs and in which they get real-time feedback that enables them to track their performance and note changes in their health quantitatively. The data they receive helps them interact more effectively with their health care providers and enables them to manage their care themselves more easily and to participate more readily in their health decisions. They also note some challenges, more so when they are acting solely on their own without the support of a clinical expert. Use of the mobile app sometimes poses a problem, especially when service is interrupted or among users less savvy about technology.

The need to provide prompt feedback to those completing a trial leads to a need to automate data cleaning and analysis to the extent possible. We have used R packages attached at the back end, either embedded within the mobile app or as a standalone program computing on data uploaded to a server (Barr et al., 2015)(Kaplan et al., 2019). The current version of the R package *nof1ins* can be found at https://github.com/jiabei-yang/nof1ins (n.d.-d). As these packages incorporate more and more sophisticated features, they should facilitate the wider adoption of personalized trials. That can only help the task of making science more approachable and more valuable to the public.

## 8. Conclusions

N-of-1 trials provide a personalized scientific approach that could greatly expand the number of people and the number of environments in which research is carried out. The Bayesian approach offers a means to incorporate participants’ own beliefs and to express results probabilistically in a way that helps participants make decisions. With flexible models and software that implements them behind the scenes and then reports results to users intuitively, Bayesian models can facilitate the spread of these tailored research designs.

## Figures and Tables

**Table 1.** Median posterior treatment difference, 95% credible interval and posterior probability of improvement (difference <0) of $\text{PROMIS} ^{\text{\tiny{\textregistered}}}$ Pain Interference for 24 girls and 30 boys using a multiple imputation model.

	Girls			Boys		Girls vs. Boys			
	
Comparison	Median	95%CrI	Pr <0	Median	95%CrI	Pr <0	Median	95%CrI	Pr <0
SCD v Baseline	−4.79	−6.65, −2.87	1.00	−1.62	−3.28, 0.00	0.97	−3.19	−5.66, −0.51	0.99
MSCD v Baseline	−4.53	−6.44, −2.56	1.00	−1.73	−3.56, 0.22	0.96	−2.87	−5.39, 0.05	0.97
SCD v MSCD	−0.27	−1.47, 0.92	0.67	0.08	−1.11, 1.25	0.45	−0.35	−2.00, 1.31	0.66

*Note* SCD – Specific Carbohydrate Diet; MSCD – Modified Specific Carbohydrate Diet; CrI – Bayesian Credible Interval.
